# Investigation of Gleditschine

**Published:** 1887-12

**Authors:** 


### INVESTIGATION OF GLEDITSCHINE.

In our last number we gave an account of a fraud which had been perpetrated on the medical profession in the alleged discovery of a new anæsthetic, called *gleditschine* and *stenocarpine.*

Dr. Shrady, the progressive editor of the *N. Y. Medical Record*, is having the matter thoroughly investigated by Dr. Charles Rice, an eminent chemist of New York. The results of his labor will soon be announced to the profession.

### ITEMS.

A MOST important question with practicing physicians is how and where they may procure the drugs and appliances in daily use in their avocation that are absolutely pure, or of at least reasonably good quality.

However well-grounded they may be in the principles of materia medica and therapeutics, they have not always at hand the chemical tests nor the chemical skill necessary to determine the purity of the remedies which they employ.

They are therefore compelled, to a great extent, to rely upon the reputation of the proprietors of certain manufacturing pharmaceutical establishments.

This reputation for honesty and fidelity is acquired by a long course of fair dealing with their customers, who have tested in their daily labors the efficacy of the supplies furnished to them, or by information furnished them in the pages of the medical journals throughout the country. Enterprising manufacturers frequently supplement this legitimate means of information by sending their agents, supplied with personal pamphlets and lists, to call the attention of physicians to particular preparations or the houses which supply them.

This may be considered fair and legitimate, and by these methods manufacturing establishments slowly attain to such repute that the supplies which they furnish are accepted as genuine without further inquiry.

Other pharmaceutical establishments, not satisfied with these recognized and usual methods of calling the attention of the public to their preparations, have undertaken the publication of so-called medical journals, pretending to advance the general literature of the profession in what are actually merely personal advertising sheets.

This mode of advertising is by many regarded as unfair to the customers of these houses, as well as to the publishers of legitimate medical journals, whose advertising pages are the usual vehicle for such information.

An advertisement in one of those, however laudatory of the manufactory for the product which it praises, carries with it only the force of an interested publication, and is no more worthy of confidence than the antiquated announcement in the label on certain bottles, that "none are genuine unless signed by old Jacob Townsend."

In fact, such publications often defeat the objects they are intended to secure. A few years since many of Dr. E. R. Squibbs' preparations stood unchallenged before the public; they were as readily prescribed and more implicitly relied upon than the officinal articles of the materia medica; but since he has chosen to sustain their reputation by the appliances above alluded to, physicians have learned that remedies of equal purity and perhaps similar cost are manufactured by other houses, and Dr. Squibbs himself will soon learn that the regular medical journals of the country still have access to the ear of the profession, and are the source through which they gain the information on which they predict their opinion, and that the only reliable medicines to be had are not those that come from the establishment of Dr. E. R. Squibbs.

LIVER SURGERY—SUCCESSFUL CHOLECYSTENTEROSTOMY.—In the *Medical Record*, of New York, for November 12th, the following data are presented: The results of the surgical operations upon the gall-bladder and the ducts of the liver have been by no means discouraging. Among thirty-three cases of cholecystotomy collected by Dr. Gaston (Wood's "Reference Handbook") there were nine deaths, or 27.27 per cent. Among the last twelve operations there was only one death. The incompleted operations have been more fatal, but their number is small. The more complicated operation of "cholecystenterostomy"—*i. e.*, of uniting the gall-bladder and intestine by a fistulous opening on account of closure of the common duct—has as yet been attempted but few times, and the best methods for its performance, as well as the nature of the results, are still undetermined. This operation, suggested by Nussbaum in 1880, was first performed by Winiwarter a year later (*Prag. Medic. Wochensch.*, 1882, No. 21 u. 22). A number of experimental observations were subsequently made by Harley, Witzel (*Deut. Zeitsch. f.*

*Chir.*, 21 Bd., S. 199, 1885) Golzi (*Centralbl. f. Chir.*, 1886, No. 24) and Gaston (*Brit. Med. Journal*, February 5, 1887). The latter author in particular devised an ingenious operation which goes by his name, but one which Kappeler considers too uncertain and dangerous to be applied to man.

Professor O. Kappeler has recently (*Correspondenzblatt f. Schweiz. Aerzte*) reported a case of permanent closure of the common bile-duct, with consequent icterus, in which he successfully united the gall-bladder to the intestine, and thus established a new path for the passage of the bile. The patient was a man fifty-five years of age, who suffered from obstructive jaundice, due, as the operation showed, to pressure on the ductus choledochus by a pancreatic tumor. The gall-bladder was greatly distended. The operation consisted of emptying the gall-bladder and stitching an opening in its walls directly to a corresponding slit in the duodenum. The operation is described as simple and safe. In any event, it was successful in Kappeler's hands. He terms it a "unilateral cholystenterosectomy."

The original experiment of Dr. Gaston for the verification of the practicability of a communication from the gall-bladder into the duodenum as a measure of relief in obstruction of the common bile-duct appeared in this journal. We are, therefore, especially interested in the favorable result of the operation by Kappeler for securing this object. It would seem that his modification of Gaston's operation must be attended with greater risk than the simple union of the walls by a single stitch of thread, as was found effectual in dogs, and hence the result may be regarded as assured in similar cases.

THE "SCHOOLMASTER" ON HIS TRIAL.—In the lectures on "The Modern Tendency of Disease," which have recently appeared in our columns, Dr. Fothergill attacks the schoolmaster from a totally novel standpoint. It does not appear that the lecturer is opposed to education generally, or is in any way an advocate for ignorance ; indeed, he is a willing teacher himself, but he sees the labors of the schoolmaster from a physiological point of view. Without endorsing all his statements, it must be admitted that he ingeniously places certain recognized facts in a very suggestive relationship. He takes up a broad ground, starting with the three layers of early fœtal development. The middle layer, the mesoblast, provides the vascular system which feeds the other layers, the epiblast and hypoblast. The outer layer, or epiblast, gives the cerebro-spinal system and the sensitive layer of the skin; or, in other words, the means by which the body is in communication with its environment. The hypoblast develops into the glandular apparatus of the viscera which open along the alimentary canal. He points out how in a primitive or uncultured race the balance of nutrition is maintained, and the child grows up a healthy animal. But the march of civilization has disturbed the balance largely by the action of the schoolmaster. His contention is that the demands of the brain "as the organ of mind" upon the nutritive middle layer tends to starve the hypoblastic structures, with the consequence that the organs of assimilation are imperfectly developed and are inadequate as to functional capacity. The dwindling of the stature in precocious town populations, he asserts is largely due to this same cause. Healthy children in the country may be equal to the synchronous demands of education and of growth; but not so town children as a rule.

Whether he is fully justified in the hypothesis he puts forward or not, the ingenuity of the argument must be admitted. He has also rendered the issue a plain one by reducing the complex subject to its simplest elements. Do the demands of education upon the budding organism disturb the natural balance of nutrition? This is an aspect of the subject which has not so far, it would appear, been placed before the school board. Of the excellence of knowledge all are agreed. The process of education must commence in tender years and be carried on during the period of growth. That is inevitable. But if a certain antagonism can be demonstrated to exist betwixt intellectual evolution and the physical development, the matter becomes serious, and must attract, as it deserves, the attention of all thinking persons. The physiologist, the schoolmaster and the philanthropist are alike interested in bringing the matter to a definite issue. Are the facts, as the lecturer states, or are his views not a real scene, and merely a phantasmagoria? It is not so very long since Sir James Crichton Browne took up the subject of overwork in board schools; and though the partisans of compulsory education assailed him vigorously, he repelled their attack most successfully. He approached the subject from the ground of what he personally encountered in his visits to the various preliminary schools. Now another member of the profession comes forward to point out the drawbacks attaching to too much education on a broad physiological basis. Of the *a priori* advantages of education no one doubts, nor can elementary education be deferred till the period of physical growth is over. The demands of modèrn life forbid the suggestion of any postponement of the educational process. But if it can be shown that the effect of excessive demand upon the brain is to disturb the nutritive balance of the body, and so to impair its physique, then it is equally clear that the present educational demands must be moderated. The matter is in the air. Not only did Dr. Milner Fothergill bring the subject of "The Effects of Town Life upon the Human Body" before the British Association at Manchester, but recently Dr. Mott contributed a paper to the *Edinburgh Medical Journal* on the subject of "Intellectual Evolution and its Relation to Physiological Dissolution," in which he pointed out how the older civilizations became effete and perished under the assault of the unlettered barbarian, the weakly man of culture being unequal to battle with the rude man of unimpaired physique. He then enumerated the forces in action upon the modern town-dweller; and he, too, arrived at the conclusion that intelluctual evolution tells injuriously upon the physique. Physique and psychique are antagonistic, it would seem. The schoolmaster seemingly entails physical degeneracy. It is a terrible conclusion if it can be substantiated, and its proof or disproof ought at once to be forthcoming. The investigation ought to be pushed without one moment's delay. The matter is superlatively important, and is neither more nor less than a national question. Of the physical deterioration of urban populations we are only too sure, and that this is by the road of reversion to an earlier and lowlier ethnic form seems highly probable. This may be inseparable from life in large towns; but if such is the case it is all the more important to ascertain what avoidable causes are in action aiding and abetting in this physical deterioration. If the charge against the schoolmaster can be substantiated, then in the interests of humanity the educational activity of to-day must be moderated and its fervor somewhat abated.

As to the further matters of the impaired digestive powers of town-dwellers, inciting them to a dietary rich in meat, and therefore favorable to the development of Bright's disease, it cannot be touched upon here. Also we cannot now discuss the matter of the relations of higher education to the development of puberty, especially in girls. But certainly Girton and Newnham Colleges must look to this matter. Education is a good thing; so no doubt is the higher education of women. But if it has to be purchased at the cost of physical degeneracy, then the price is too great.

Brains are the finest raw product of any country, and ought to be made the most of. But as God tempers the wind to the shorn lamb, so the poor, under-fed town-mite ought to be considered in our compulsory education. What may be excellent for strong children may be burdensome to the weakly, and our teachers should not impose upon delicate and under-fed children burdens heavier than they can bear.

HOMŒOPATHY.—Our Liverpool correspondent sends us a morning journal of that city containing an account of a new homœopathic hospital, together with a leader on the relations of that part of the medical profession styled "allopaths" by a portion of the publie and by those who call themselves "homœopaths" with the minute section who assume the latter title. The article in question shows gross ignorance of a subject on which the writer assumes himself to be in a position to lecture others, but appearing as it does in the columns of a respectable journal, it ought not to pass unchallenged. Near the commencement of the article the writer, speaking of the new era, says: "Homœopathy will enter upon a still more vigorous competition with allopathy."

Now, it should ever be borne in mind by ourselves, and even inculcated at every opportunity upon others, that the designation, "allopath," is one bestowed by the self-styled "homœopaths" to suit their own party purposes. It is one not recognized by practitioners of legitimate medicine, but is repudiated with almost as much contempt as we should repudiate its antithesis. Not only do we reject the nickname—for it is nothing more—heartily, but authority is against our assumption of it, even if we were so disposed. The highest medical authority, in England at least, will be generally recognized to be the Royal College of Physicians, and on December 27th, 1881, this body, after mature deliberation, passed a resolution bearing on this very point, which speaks out with as much decision as it is possible to put into words. The resolution is as follows: "That while the college has no desire to fetter the opinion of its members in reference to any theories they may see fit to adopt in connection with the practice of medicine, it nevertheless considers it desirable to express its opinion that the assumption or acceptance by members of the profession of designations implying the adoption of special modes of treatment is opposed to those principles of the freedom and dignity of the profession which should govern the relations of its members to each other and to the public." Having delivered its judgment, the college goes on to state what it expects from those under its authority: "The college therefore expects that all its fellows, members and licentiates will uphold these principles by discountenancing those who trade upon such designations." It is thus plain that the college does not countenance the assumption or acceptance of the designation of "allopath," and that such an assumption or acceptance would land us in the same condemnation with the "homœopaths." After this repudiation we expect that "homœopaths" will call us "allopaths" because it suits their trade purposes to do so, but we also expect that the intelligent public will refrain from the use of a designation which it is contrary to authority for us to assume or accept, and which we indignantly repudiate. Not only do we repudiate the name, but we repudiate also the principles of allopathy, or rather we ignore them. We do not profess to know anything of them. They have never been our principles.

We have been accused by homœopaths of holding these principles, but we assure both them and the public that the accusation is false. Our only aim is to relieve suffering and prolong life, and we ask the public to believe us when we assert that in our estimation the question whether the methods we employ are allopathic or homœopathic is so far beneath us as not to be deserving of a thought. So far the nick-name "allopath." The writer goes on to congratulate himself and his readers that the poor will at last have the "great boon" of hospital treatment by homœopathic practitioners. We do not for a moment doubt that the poor will be well treated in Mr. Tait's new hospital, but we do strongly doubt that they will get "homœopathic" treatment when the diseases are serious or well-pronounced. It is not so much the ability to cure as the *bona fides* of the professed "homœopath' that we doubt. It goes without saying that in the new hospital, as in all their practice, active drugs will be requisitioned by the homœopaths and claimed as theirs and used in the treatment of disease. Heart disease will be treated by rest, digitalis, convallaria, coffee or strophanthus; acute rheumatism by salicylates in some form; skin diseases by arsenic; phthisis by the hypophosphites and cod-liver oil; hæmoptysis by lead and gallic acid or ergot; lead-poisoning and syphilis by iodide of potassium; and so on through the whole materia medica. It is notorious that all these drugs are non-homœopathic, and that they are in regular use amongst homœopathic practitioners; that they, in fact, cure whatever cases they do cure by non-homœopathic means, and claim the credit for their so-called "system." It is not honest, and this is the only quarrel we have with them. Only recently a gentleman suffering from lead palsy was treated, "homœopathically," of course, by eight-grain doses of potassic iodide thrice daily in water, but then he had some pilules as well, and also, of course, it was the pilules, and not the iodide, that cured the disease. This is an authentic case, but such could be multiplied indefinitely.—*Medical Press and Circular.*

